# Supplementary material for: TRIM37-mediated stabilization of PEX5 via monoubiquitination attenuates oxidative stress and demyelination in multiple sclerosis insights from EAE and LPC-induced experimental models
Source: PLoS One. 2025 Oct 24;20(10):e0335198. doi: 10.1371/journal.pone.0335198 (PMC12551917; doi:10.1371/journal.pone.0335198)
Supplement: S1 File — (PDF) [file pone.0335198.s001.pdf]

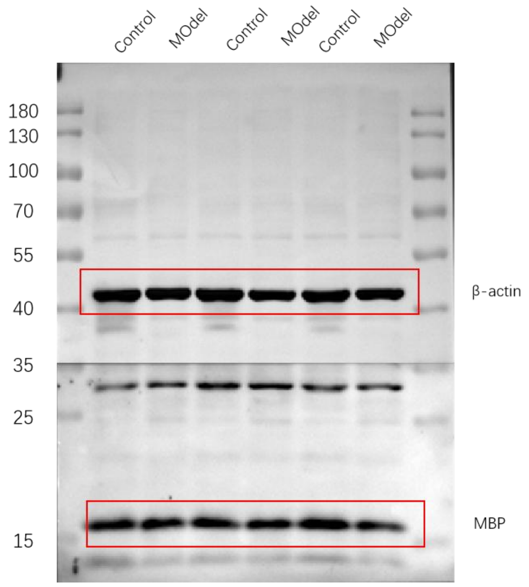

S1 Fig. Western Blot full-length images of Figure 3a. The images include the complete electrophoretic gel to ensure the integrity of protein bands and the accuracy of loading controls.

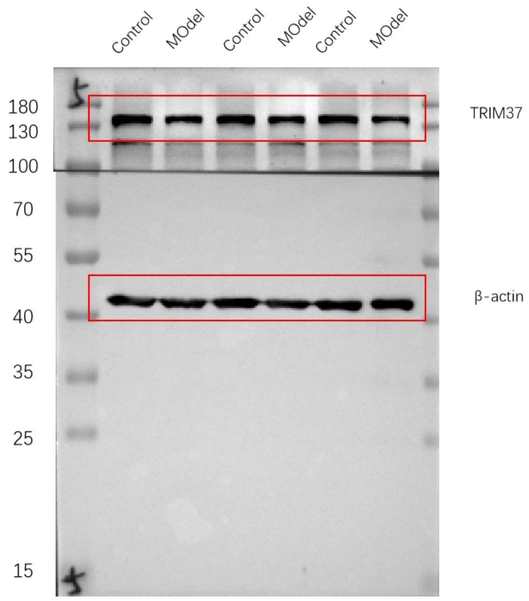

S2 Fig. Western Blot full-length images of Figure 3i. The images include the complete electrophoretic gel to ensure the integrity of protein bands and the accuracy of loading controls.

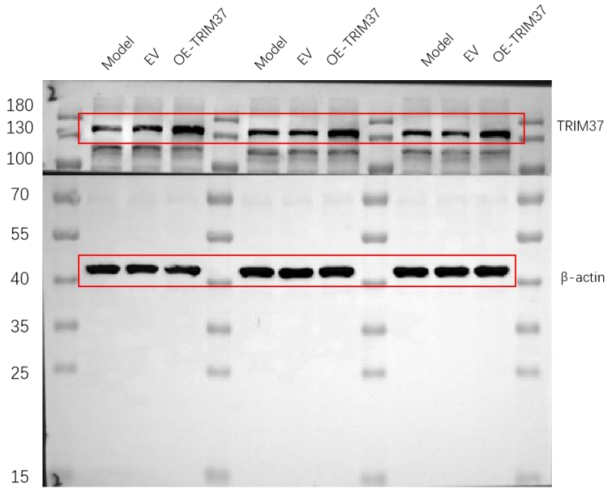

S3 Fig. Western Blot full-length images of Figure 4b. The images include the complete electrophoretic gel to ensure the integrity of protein bands and the accuracy of loading controls.

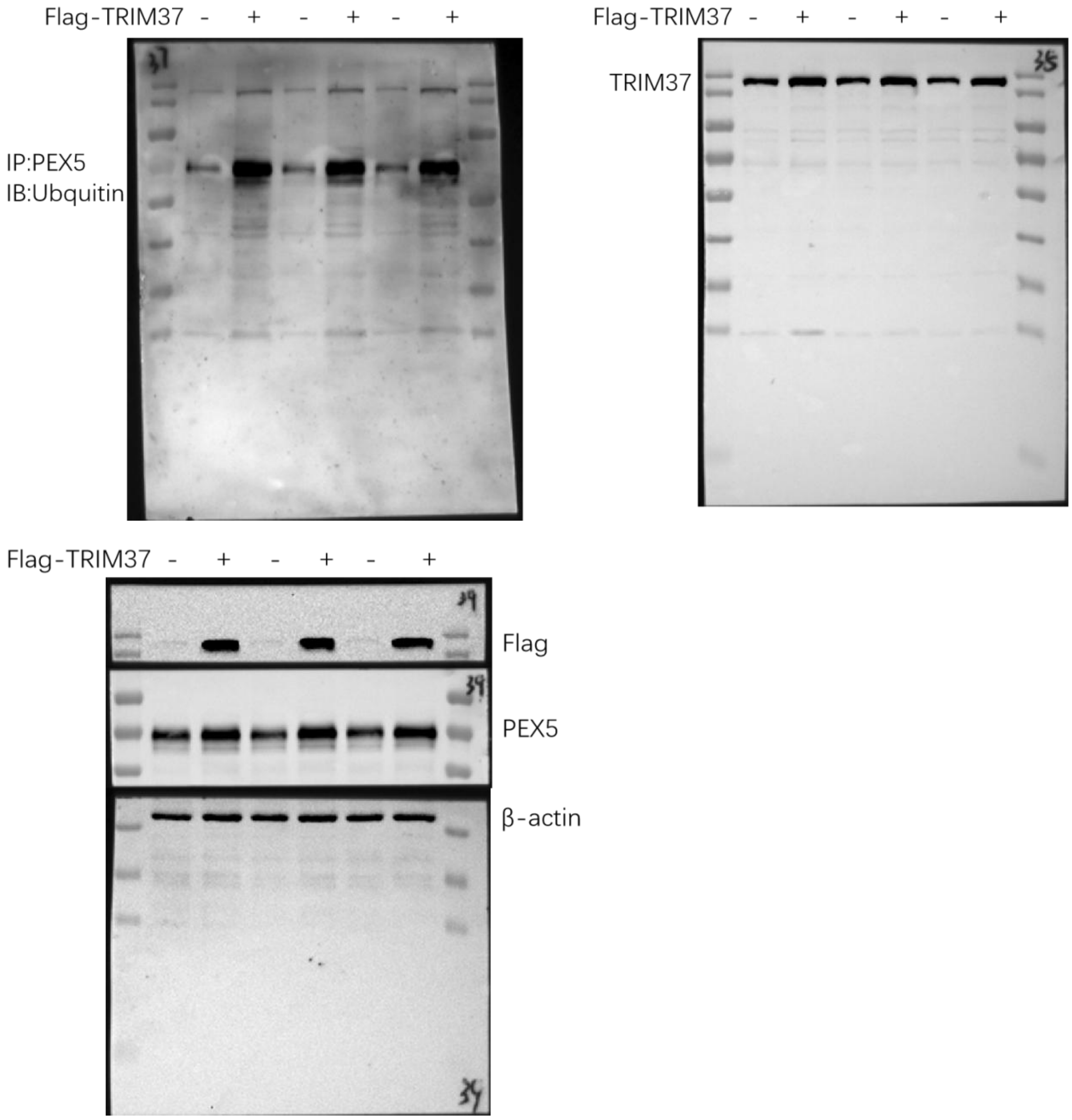

S4 Fig. Western Blot full-length images of Figure 4d. The images include the complete

electrophoretic gel to ensure the integrity of protein bands and the accuracy of loading controls.

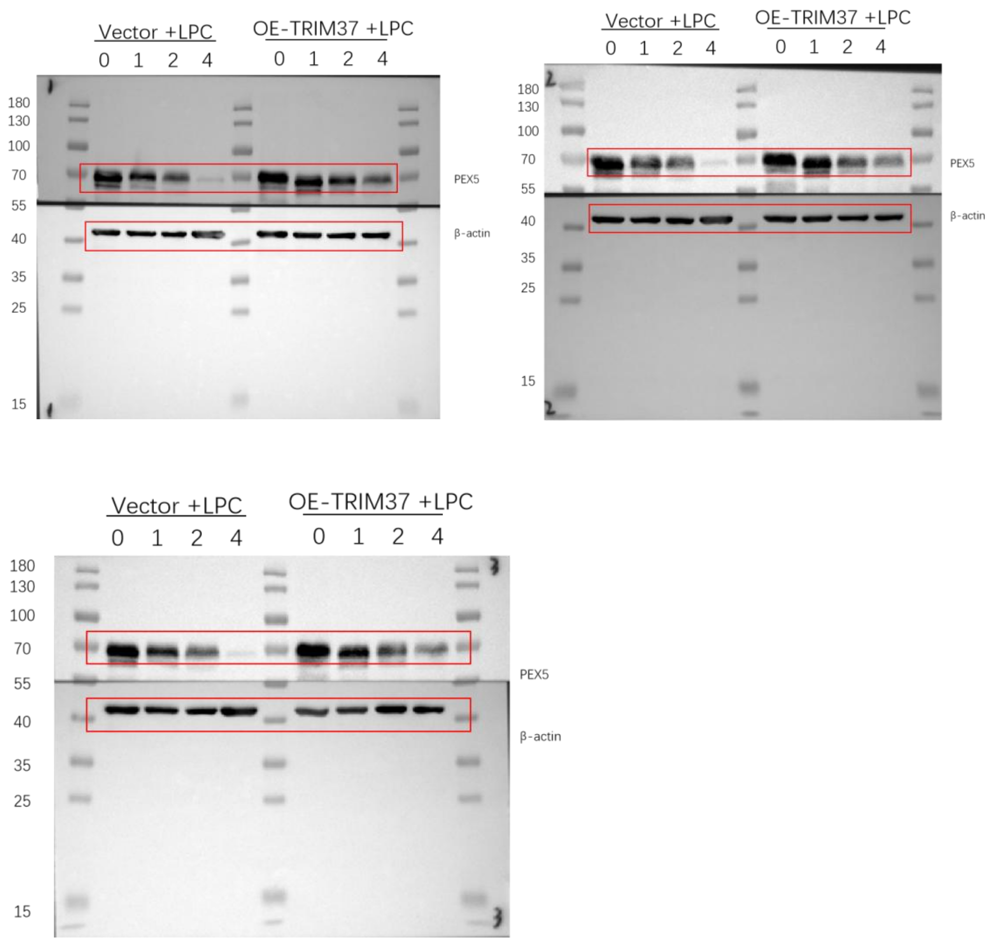

S5 Fig. Western Blot full-length images of Figure 4g. The images include the complete electrophoretic gel to ensure the integrity of protein bands and the accuracy of loading controls.

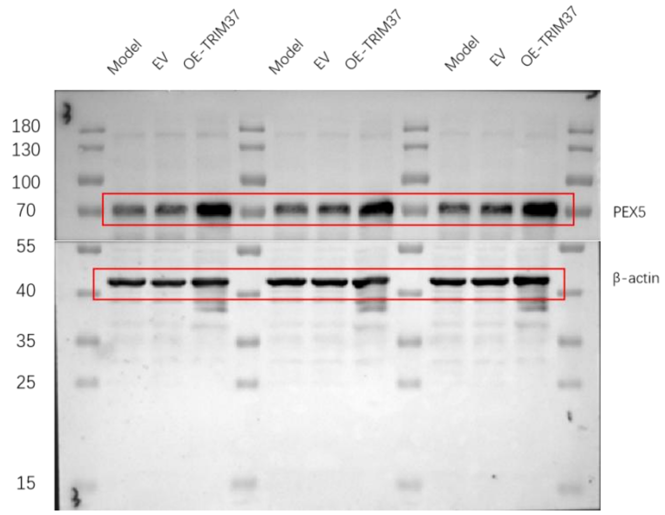

S6 Fig. Western Blot full-length images of Figure 5a. The images include the complete electrophoretic gel to ensure the integrity of protein bands and the accuracy of loading controls.

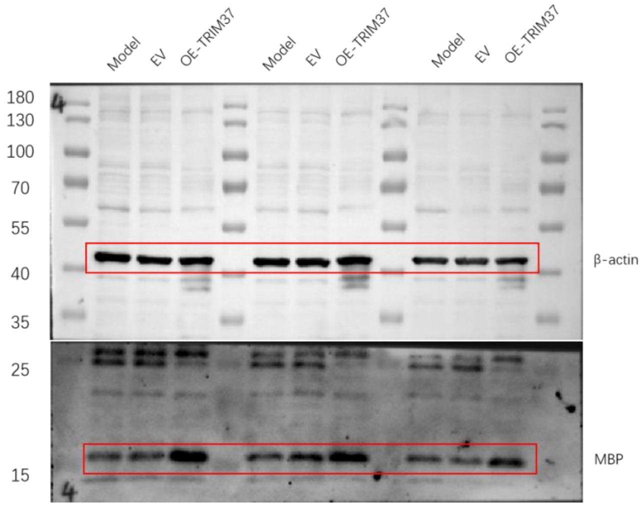

S7 Fig. Western Blot full-length images of Figure 5g. The images include the complete electrophoretic gel to ensure the integrity of protein bands and the accuracy of loading controls.
