# Supplementary material for: TRIM37-mediated stabilization of PEX5 via monoubiquitination attenuates oxidative stress and demyelination in multiple sclerosis insights from EAE and LPC-induced experimental models
Source: PLoS One. 2025 Oct 24;20(10):e0335198. doi: 10.1371/journal.pone.0335198 (PMC12551917; doi:10.1371/journal.pone.0335198)
Supplement: Attachment — Submitted filename: Review PLOS ONE.docx [file pone.0335198.s003.docx]

PLOS ONE

TRIM37-Mediated PEX5 Stabilization via Monoubiquitination Alleviates Oxidative

Stress and Demyelination in Multiple Sclerosis-REVIEW

1. The study presents the results of original research.

The topic is original and no one has ever investigated the TRIM37-PEX5 axis in this way. TRIM37 is reduced in MS patients and its importance is reflected in its participation in the functioning of peroxisomes and its contribution to the development of oxidative stress. The current topic is the study of the inhibition of apoptosis of neurons and glial cells and the stimulation of remyelination.

2. Results reported have not been published elsewhere.

The authors link the TRIM37-PEX5 axis to the pathogenesis of MS for the first time, contributing to new insights into pathophysiology and potential therapeutic directions.

3. Experiments, statistics, and other analyses are performed to a high technical standard and are described in sufficient detail.

This is a detailed and extremely informative paper written according to all technical standards. The authors used bioinformatics methods, human cell cultures, polymerase chain reaction with reverse transcription to detect the level of messenger RNA, Western blot, immunofluorescence, they detected the level of free radicals and cellular apoptosis. To detect the effect of TRIM37 on PEX5 ubiquitination, they used co-immunoprecipitation with Western blot technique and CHX method to detect the half-life of the protein. Additionally, they examined TRIM37 expression in an animal model of experimental autoimmune encephalomyelitis. The corpus callosum was processed by histological techniques (hematoxylin and eosin, Luxol fast blue) and immunohistochemical methods. Statistics were correctly performed. It is commendable that each procedure was processed in detail.

4. Conclusions are presented in an appropriate fashion and are supported by the data.

The genes identified in this study may influence the progression of MS. TRIM37 plays a pivotal role in the pathogenesis of MS. Downregulation of TRIM37 is accompanied by increased oxidative stress and cell apoptosis in an in vitro model. Increased expression of TRIM37 leads to monoubiquitination of PEX5, which exerts a protective effect. The role of TRIM37 in stimulating myelination and inhibiting inflammatory cell infiltration has been demonstrated. This is the first in vivo evidence that the TRIM37-PEX5 axis acts dually by both repairing myelin and influencing the inflammatory environment. This may be a potential therapeutic target.

5. The article is presented in an intelligible fashion and is written in standard English.

The manuscript is well structured, with a clear abstract, introduction, methodology, results and discussion. The language is technically precise and clear, and figures and graphs support the statements in the text.

6. The research meets all applicable standards for the ethics of experimentation and research integrity.
1. Authors respected ARRIVE guidelines and the Animal Ethics Committee of Beidahuang Group General Hospital approved this research. The use of anesthesia and euthanasia in animal models was carried out according to rules and guidelines. Тhe Health Commission of Heilongjiang Province funded this research without any influence on the research. The methodology is sound, experiments were replicated, appropriate controls were used, and statistical analyses were correctly applied. The discussion also acknowledges limitations, which strengthens the overall reliability of the findings.
7. The article adheres to appropriate reporting guidelines and community standards for data availability.

All relevant data are made available within the manuscript and supplementary files, in accordance with open access data policies and good scientific practice.

The study meets all ethical and integrity standards required for publication in a peer-reviewed scientific journal.

With respect,

Dr med Simonida Delic	16^th^ of July,2025.
