# Supplementary material for: TRIM37-mediated stabilization of PEX5 via monoubiquitination attenuates oxidative stress and demyelination in multiple sclerosis insights from EAE and LPC-induced experimental models
Source: PLoS One. 2025 Oct 24;20(10):e0335198. doi: 10.1371/journal.pone.0335198 (PMC12551917; doi:10.1371/journal.pone.0335198)
Supplement: Attachment — Submitted filename: Response to Reviewers.docx [file pone.0335198.s005.docx]

Dear Editor,

Thank you very much for your valuable feedback and the opportunity to revise our manuscript (PONE-D-25-33250). We greatly appreciate the time and effort the editorial team and reviewers have dedicated to evaluating our work.

We would like to inform you that all comments from Reviewer #1 and Reviewer #3, as well as the additional requirements specified by the editorial office, have been addressed in the revised manuscript. In accordance with the guidelines, we have uploaded the following files: 'Response to Reviewers', 'Revised Manuscript with Track Changes', 'Manuscript', and the 'cover letter'. Additionally, the supporting information, named "S1_raw_images", has been uploaded as required.

All requested revisions have been implemented, and the changes made are clearly visible in the 'Revised Manuscript with Track Changes' for easy reference.

We sincerely appreciate the valuable feedback and guidance provided by the editorial team and reviewers, which have significantly improved the quality of our manuscript.

**Below is a point-by-point response to the additional requirements outlined by the editorial office：**

**1. Please ensure that your manuscript meets PLOS ONE's style requirements, including those for file naming. The PLOS ONE style templates can be found at https://journals.plos.org/plosone/s/file?id=wjVg/PLOSOne_formatting_sample_main_body.pdf and https://journals.plos.org/plosone/s/file?id=ba62/PLOSOne_formatting_sample_title_authors_affiliations.pdf**

Thank you for your reminder regarding the manuscript formatting requirements. We have carefully reviewed the style guidelines provided in the two links: PLOS ONE Formatting Sample Main Body and PLOS ONE Formatting Sample Title, Authors, Affiliations.

Following the guidelines from the first link (Main Body Formatting Guidelines), we have adjusted the manuscript structure and formatting as required. Specifically, Level 1 headings (e.g., Abstract, Introduction, Materials and Methods) are formatted in bold with 18pt font; Level 2 subheadings are in bold with 16pt font; and Level 3 subheadings are in bold with 14pt font. All headings and titles are written in sentence case (capitalizing only the first word, the first word of subheadings, proper nouns, and genus names). Additionally, we have ensured correct citation formats for figures (e.g., "Fig 1", "Figs 1 and 2"), tables (e.g., "Table 1"), and supporting information (e.g., "S1 Fig"), with all citations appearing in numerical order at first mention.

In accordance with the second link (Title, Authors, Affiliations Guidelines), we have revised the title page to meet the specified standards. Author affiliations are listed in full order from smallest to largest organizational unit (Department, Division, Institution, City, State, Country) without abbreviations (e.g., "Department" instead of "Dept"), ZIP codes, or position titles (e.g., Professor). Symbols for contributorship are correctly applied: the corresponding author is indicated with *.

The revised manuscript with tracked changes has been named "Revised Manuscript with Track Changes" as required, with all modifications clearly presented in the revision format.

We hope these adjustments fully comply with PLOS ONE’s style requirements. Thank you for your time and guidance.

**2. PLOS ONE now requires that authors provide the original uncropped and unadjusted images underlying all blot or gel results reported in a submission’s figures or Supporting Information files. This policy and the journal’s other requirements for blot/gel reporting and figure preparation are described in detail at https://journals.plos.org/plosone/s/figures#loc-blot-and-gel-reporting-requirements and https://journals.plos.org/plosone/s/figures#loc-preparing-figures-from-image-files. When you submit your revised manuscript, please ensure that your figures adhere fully to these guidelines and provide the original underlying images for all blot or gel data reported in your submission. See the following link for instructions on providing the original image data: https://journals.plos.org/plosone/s/figures#loc-original-images-for-blots-and-gels.**

**In your cover letter, please note whether your blot/gel image data are in Supporting Information or posted at a public data repository, provide the repository URL if relevant, and provide specific details as to which raw blot/gel images, if any, are not available. Email us at plosone@plos.org if you have any questions.**

Thank you for your guidance regarding the requirements for original blot and gel images. We have carefully reviewed the guidelines outlined in the provided links, including “Blot and Gel Reporting Requirements” and “Original Images for Blots and Gels”, and have ensured full compliance with these policies.

In accordance with the first link’s instructions, we have compiled all original, uncropped, and minimally adjusted blot/gel images supporting the results in the main figures and supplemental figures into a single PDF file. This file has been named **“S1_raw_images”** and uploaded as a Supporting Information file. Each image within the file is clearly annotated to indicate the loading order, sample identities, image capture methods, and the corresponding figure panels derived from them. Molecular weight markers are included where applicable, and any lanes not featured in the final figures are marked with an “X” above their labels, with all annotations avoiding obscuration of data or background bands.

Following the guidelines in the third link, we have ensured strict adherence to proper blot/gel reporting standards: all image adjustments (e.g., brightness or contrast) were uniformly applied to entire images without altering the visibility of data or background bands; figures were not overcropped, retaining relevant background areas and non-specific bands (with explanations provided in figure legends where necessary); comparative analyses were performed using samples run on the same gel/blot; controls were included on the same blot/gel as experimental samples; and any lane rearrangements within a single blot/gel were clearly denoted by vertical black lines in the figures, with details provided in the legends. No composite images from different blots, gels, or exposures were spliced together, and separate panels were used where data from multiple sources were required.

Additionally, we confirm that our original images do not contain issues highlighted in the example of inappropriate manipulations, such as deleting/adding bands, using tools to artificially “clean up” backgrounds, or misrepresenting gel lanes through undisclosed splicing. All adjustments were made to enhance clarity while preserving the integrity of the original data.

The Supporting Information section in the manuscript has been updated to include the caption for “S1_raw_images” and in-text citations to reference this file. Additionally, we have noted in the cover letter that the blot and gel images have been included in the Supporting Information.

Please let us know if further details or adjustments are needed. Thank you for your time and assistance.

**3. PLOS requires an ORCID iD for the corresponding author in Editorial Manager on papers submitted after December 6th, 2016. Please ensure that you have an ORCID iD and that it is validated in Editorial Manager. To do this, go to ‘Update my Information’ (in the upper left-hand corner of the main menu), and click on the Fetch/Validate link next to the ORCID field. This will take you to the ORCID site and allow you to create a new iD or authenticate a pre-existing iD in Editorial Manager.**

Thank you for your reminder regarding the ORCID iD requirement for the corresponding author. We confirm that the corresponding author has completed the ORCID iD validation process in Editorial Manager as instructed. Specifically, the corresponding author accessed the "Update my Information" section from the upper left-hand corner of the main menu, clicked on the "Fetch/Validate" link next to the ORCID field, and successfully authenticated the pre-existing ORCID iD through the ORCID site. The ORCID iD has now been validated in the system.

**4. Your ethics statement should only appear in the Methods section of your manuscript. If your ethics statement is written in any section besides the Methods, please move it to the Methods section and delete it from any other section. Please ensure that your ethics statement is included in your manuscript, as the ethics statement entered into the online submission form will not be published alongside your manuscript.**

Thank you for your reminder regarding the ethics statement. We have deleted the original standalone "Ethical Statement" section and incorporated the relevant content into the "MS animal model" subsection within the "Materials and methods" section. The ethics statement now specifies that all animal experiments were conducted in accordance with the ARRIVE guidelines and approved by the Animal Ethics Committee of Beidahuang Group General Hospital (approval number: KY2024092003), ensuring compliance with the journal’s requirements.

**5. Thank you for stating the following in the Acknowledgments Section of your manuscript:**

**This research was supported by the 2024 Science and Technology Plan of the Health Commission of Heilongjiang Province (Grant No.: 20240303070234). We would like to extend our sincerest gratitude to the Health Commission of Heilongjiang Province and its relevant administrative departments. The successful execution and outcome of this project would not have been possible without your strategic support and scientific management for medical research.**

**We note that you have provided funding information that is not currently declared in your Funding Statement. However, funding information should not appear in the Acknowledgments section or other areas of your manuscript. We will only publish funding information present in the Funding Statement section of the online submission form.**

**Please remove any funding-related text from the manuscript and let us know how you would like to update your Funding Statement. Currently, your Funding Statement reads as follows:**

**The research work presented in this article was supported by the 2024 Science and Technology Plan of the Health Commission of Heilongjiang Province. The project number is 20240303070234, with Jiang Lai serving as both the project leader and the recipient of the grant. The grant was awarded by the Health Commission of Heilongjiang Province.**

**Please include your amended statements within your cover letter; we will change the online submission form on your behalf.**

Thank you for your reminder regarding funding information. We have removed all funding-related text from the Acknowledgments section of the manuscript. In the cover letter, we have updated the Funding Statement to include: "This study was supported by the Heilongjiang Provincial Health Commission Science and Technology Program (Grant No. 20240303070234). The grant was awarded to the first author, Jiang Lai, to facilitate the study. The funders had no role in study design, data collection and analysis, decision to publish, or preparation of the manuscript. This study involves no commercial cooperation. All listed authors in this study did not receive salaries or profits from any other institutions or commercial organizations. Moreover, aside from the authors listed in the manuscript, no other sponsors or funders have played any role in this study." Thank you for your assistance in updating the online submission form on our behalf.

**6. Please include captions for your Supporting Information files at the end of your manuscript, and update any in-text citations to match accordingly. Please see our Supporting Information guidelines for more information: http://journals.plos.org/plosone/s/supporting-information.**

Thank you for your valuable feedback. We have now included captions for all Supporting Information files at the end of the manuscript under the heading "**Supporting information**", as specified in PLOS ONE guidelines. Each SI figure caption describes the full-length Western blot images corresponding to the main figures (e.g., **S1 Fig** for **Fig 3a**). Additionally, we have added in-text citations to these SI files at their first mention in the Results section (e.g., "**Fig 3a, S1 Fig**").

**7. If the reviewer comments include a recommendation to cite specific previously published works, please review and evaluate these publications to determine whether they are relevant and should be cited. There is no requirement to cite these works unless the editor has indicated otherwise.**

Thank you for your reminder regarding the citation of previously published works. We have carefully reviewed all reviewer comments and confirmed that none of the reviewers recommended citing specific previously published works. Therefore, no additional citations have been added to the manuscript, and the current citations remain in line with the journal’s requirements.

**Below is a point-by-point response to the comments raised by Reviewer #1：**

1) **Title and key words must include that results are from experimental models**

**EAE model; lysophosphatidylcholine (LPC)-induced model;**

Thank you for your valuable comment that the title and key words must include that results are from experimental models. We have revised the title to "TRIM37-mediated stabilization of PEX5 via monoubiquitination attenuates oxidative stress and demyelination in multiple sclerosis insights from EAE and LPC-induced experimental models

" to explicitly indicate the experimental models used. In the key words section, we have supplemented **EAE model, LPC-induced model"** to further clarify that the in vivo studies were based on the experimental autoimmune encephalomyelitis (EAE) model and the in vitro studies utilized the lysophosphatidylcholine (LPC)-induced model. These revisions aim to make the experimental background of the study more transparent.

2) **ine 41 a reference is needed after age range**

Thank you for your valuable comment regarding the need for a reference after the age range in Line 41. We have supplemented the citation as suggested. Specifically, after the description of the age range ("young adults aged 18-40") in Line 41, we have added a citation to the study by Jakimovski, D. et al. (2024). This reference was previously listed as Reference 3 and has now been updated to Reference 1 in the revised manuscript to ensure consistency with the citation order.

3) **Line 86 Caps are missing**

Thank you for your comment regarding "Line 86 Caps are missing". Upon careful review of the manuscript, we confirm that Line 86 is an empty line intentionally used to separate paragraphs, as part of our formatting practice to insert a blank line after the end of each paragraph for readability. Since there is no text in Line 86, there are no letters requiring capitalization. We have also thoroughly checked other lines in the manuscript to ensure proper capitalization of terms (e.g., proper nouns, acronyms) and found no additional issues related to missing capitalization.

**4) Line 332 use subscript number for peroxide**

Thank you for your valuable comment regarding "Line 332 use subscript number for peroxide". We have revised the text as suggested: the formula for hydrogen peroxide has been updated to "H₂O₂" with the numbers formatted as subscripts.

**Below is a point-by-point response to the comments raised by Reviewer #3：**

1) **The context is heavily focused on the pathophysiology of MS, but no background has been provided regarding TRIM37 and peroxidation in relation to Multiple Sclerosis. Therefore, it is unclear what has previously been described and why this particular protein was chosen over others.**

Thank you for your valuable comment highlighting the lack of background on TRIM37 and peroxidation in relation to MS. We have addressed this by supplementing relevant background content in the Introduction section, specifically after the discussion of TRIM37’s basic function and peroxisomal role, and before outlining the rationale for the current study. The added content clarifies the link between peroxisomal dysfunction, oxidative stress, and MS pathogenesis, summarizes existing knowledge on TRIM37’s regulation of peroxisomal function, and explains why TRIM37 was selected as a target based on its potential role in MS-related oxidative stress and demyelination. These revisions aim to provide a clearer context for the study’s focus.

2) **The study objectives have not been outlined.**

Thank you for your insightful comment regarding the clarification of study objectives. We have now added a dedicated paragraph at the end of the Introduction section to explicitly outline the four primary aims of this study:

(1) Validating TRIM37’s role in MS pathogenesis;

(2) Mechanistically linking TRIM37-PEX5 monoubiquitination to peroxisomal dysfunction;

(3) Evaluating the axis’s therapeutic potential against oxidative stress and demyelination;

(4) Establishing TRIM37 as a novel target via in vitro/in vivo models.

This addition strengthens the focus and provides clear direction for the subsequent methodology and results. We appreciate your guidance in improving the manuscript’s clarity.

3) **The methodology is confusing and appears to be disorganized.**

Thank you for your valuable comment regarding the confusion and disorganization in the methodology section. We have revised the structure of the methodology to improve clarity, as follows:

We have reorganized the original 15 methodological components into a three-tiered framework, with the first tier consisting of 7 main sections: **Bioinformatics Analysis**, **In Vitro Model Establishment**, **Genetic Manipulation**, **Molecular Analyses**, **Functional Assays**, **In Vivo Validation**, and **Data Statistics**.

The positions of the original "Bioinformatics Analysis" and "Data Statistics" sections remain unchanged.

Other components have been reclassified:

"Cell Culture" and "MS In Vitro Model Induction" are now included under **In Vitro Model Establishment**.

"Oligodendrocyte Overexpression TRIM37" is grouped into **Genetic Manipulation**.

"qRT-PCR", "Western Blot", "Immunofluorescence", and "Co-Immunoprecipitation (Co-IP)" are categorized under **Molecular Analyses**.

"Reactive Oxygen Species (ROS) Detection", "TUNEL Cell Apoptosis Detection", and "CHX Method for Protein Half-Life Detection" are included in **Functional Assays**.

"EAE Model Establishment" and "Histopathological Staining" (which encompasses "HE Staining", "LFB Staining", and "Immunohistochemistry") are now part of **In Vivo Validation**.

These revisions aim to enhance the logical flow and organization of the methodology section.

4) **Although the results are statistically significant, the conclusions and the implications for MS—both physiopathologically and therapeutically—are exaggerated and inconsistent with the findings. It would be advisable to moderate these conclusions.**

Thank you for your valuable feedback on moderating the conclusions. We have revised the **Discussion and Conclusion sections** to more accurately reflect the study's scope:

**Replaced definitive claims** (e.g., "offers a new direction") with **qualified language** ("may offer a	potential direction," "warrants further investigation").

**Explicitly framed EEA model findings as preliminary**, emphasizing **"demonstrated in the EAE	model"** and **"clinical translatability requires further validation"**.

**Added cautionary statements** in the Conclusion regarding **pathophysiological/therapeutic	implications** (e.g., "represents a candidate target," "future work is needed").
These changes ensure conclusions align with the experimental evidence while maintaining scientific rigor.

5) **The limitations section does not include other relevant factors, such as those inferred from the bioinformatic analysis or the lack of assessment of additional oxidative stress parameters.**

Dear Reviewer, thank you for highlighting these important limitations. We have expanded the Limitations section in the Discussion by adding two key points: (1) **The lack of functional validation for additional candidate genes/pathways identified in bioinformatic analysis (e.g., mitochondrial respiratory chain complex and macroautophagy-related genes) and their potential interactions with the TRIM37-PEX5 axis**; (2) **The incomplete assessment of oxidative stress parameters beyond ROS levels, including glutathione, antioxidant enzymes (SOD, CAT), and lipid peroxidation markers**. These additions provide a more comprehensive critique of the study's boundaries.

We believe these revisions have significantly improved the clarity, rigor, and completeness of our manuscript. Thank you again for your guidance. We look forward to your further evaluation.

Sincerely,

Jin Fu

E-mail: fujin@hrbmu.edu.cn
